# Combined test of third lumbar skeletal muscle index and prognostic nutrition index improve prognosis prediction power in resected colorectal cancer liver metastasis

**DOI:** 10.18632/aging.102457

**Published:** 2019-11-22

**Authors:** Yang Lv, Mei-Ling Ji, Qing-Yang Feng, De-Xiang Zhu, Song-Bin Lin, Yi-Hao Mao, Yu-Qiu Xu, Peng Zheng, Guo-Dong He, Jian-Min Xu

**Affiliations:** 1 Department of General Surgery, Zhongshan Hospital, Fudan University, Shanghai 200032, China; 2 Department of General Surgery, Zhongshan Hospital Xiamen Branch, Fudan University, Xiamen 361000, China

**Keywords:** colorectal cancer liver metastasis, skeletal muscle index, prognostic nutrition index, prognosis

## Abstract

Background: In this paper, we aim to explore clinical value of skeletal muscle index (SMI) and prognostic nutrition index (PNI) on resected colorectal cancer liver metastasis (CRLM).

Results: Among the 539 patients, 355 were males. Baseline lower SMI was associated with smaller BMI, smaller PNI, smaller pre-albumin and longer hospitalization days (P<0.05). Patients with lower SMI and PNI had significantly shorter duration of PFS and OS (P<0.05). SMI can reflect the postoperative treatment response. Postoperative 6-month’s and 12-month’s SMI and PNI can indicate overall prognosis. When combined SMI and PNI, prognostic AUC of ROC curves improved significantly.

Conclusion: Combined monitor of SMI and PNI can improve the power at predicting prognosis. Postoperative 6-month’s record of SMI and PNI was more accurate and predictive for CRLM prognosis.

Method: A total of 539 resected CRLM patients between January 2013 to December 2016 with complete clinical data were included. Computed tomography image was collected from each patient. Receiver-operating characteristic (ROC) curves were constructed; area under curves (AUC) were also determined. All clinical variables were analyzed in proper way.

## INTRODUCTION

Colorectal cancer (CRC) is one of the most common malignancies of digestive gastrointestinal tract [1, 2]. In China, approximately 20% to 25% of CRC patients have liver metastases (CRLM) at the time of initial diagnosis and overall prognosis is poor [3]. Resection is now considered as the optimal method to improve CRLM patients’ survival [4]. Thus effective markers affecting postoperative prognosis are still warranted.

For CRLM, it is still the regular circulating tumor biomarkers (CEA and CA19-9) and radiological examination (CT: computed tomography and MRI: magnetic resonance imaging) that determine treatment decisions and predict prognosis. Besides, many body-composition markers, such as skeletal muscle index (SMI) and prognostic nutrition index (PNI) have been reported to be associated with survival, complication and hospitalization in patients [5–8]. With regard to these factors, loss of SMI has been proven to be significantly present in cancer patients with distant metastasis [9]. PNI, a known marker reflecting circulating albumin level and lymphocyte number, was also proven to be predictive in cancer patients [10, 11].

So far, combined effects of SMI and PNI for resected CRLM patients has not been demonstrated. Thus in this study, we aim to explore combined clinical value of SMI and PNI in resected CRLM patients.

## RESULTS

### Patients’ common characteristics and survival

539 patients with CRLM were recruited in this study. Mean (SD) age was 60.6 (±11.7) years old, ranging from 24 to 92, and 355 (65.9%) patients were male. Univariate and multivariable Cox regression analysis for OS are outlined in Table 1. Higher T stage, higher N stage, lower differentiation grade and microvascular involvement were significantly associated with worse outcomes (all P-value<0.05). Besides, patients received simultaneous resection of liver metastasis demonstrated better survival outcomes (P-value<0.01). Interestingly, there were no statistically significant differences between study participants in regard to gender, age, primary tumor location and peri-neural invasion in our series (P-value>0.05). From multivariable analysis, we found that T stage (P-value=0.02), tumor differentiation (P-value=0.01), Baseline SMI level (P-value<0.01) and simultaneous metastasectomy status (P-value<0.01) were regarded as independent risk indicators for OS.

**Table 1:** Association of overall survival with clinicopathological characteristics in colorectal cancer patients.

**Characteristics**	**N**	**Univariate analysis**			**Multivariate analysis**		
		**HR**	**95% CI**	**P-value**	**HR**	**95% CI**	**P-value**
Ages (years)				0.29			0.12
< 60	224	1.00			1.00		
≥ 60	315	1.33	0.78-2.26		1.35	0.82-1.93	
Gender				0.77			0.89
Females	184	1.00			1.00		
Males	355	1.08	0.64-1.83		1.01	0.56-1.74	
Location				0.45			0.52
Left sided colon	232	1.00			1.00		
Right sided colon	121	1.23	0.78-1.45		1.12	0.63-1.48	
Rectum	186	0.89	0.65-1.23		0.92	0.55-1.28	
T stage				0.02			0.02
T1/T2	87	1.00			1.00		
T3/T4	452	2.32	1.86-2.66		2.02	1.52-2.42	
N stage				<0.01			0.05
N0	57	1.00			1.00		
N1/N2	482	1.89	1.65-2.13		1.23	0.89-1.67	
Differentiation				<0.01			0.01
Low grade	352	1.00			1.00		
High grade	187	2.01	1.45-2.23		1.62	1.48-1.92	
Microvascular involvement				<0.01			0.456
Absent	100	1.00			1.00		
Present	439	1.44	1.12-1.65		1.21	0.68-1.41	
Peri-neural invasion				0.08			0.06
Absent	221	1.00			1.00		
Present	318	1.22	0.91-1.35		1.34	0.89-1.43	
Simultaneous Metastasectomy				<0.01			<0.01
Performed	181	1.00			1.00		
Not performed	358	2.45	1.66-2.89		2.22	1.87-2.43	
Baseline SMI level				<0.01			<0.01
<43 (men), <41 (women)	309	1.00			1.00		
≥43 (men), ≥41 (women)	230	1.73	1.19-2.52		1.63	1.29-2.32	

### Correlation of baseline physiological compositions and clinical characteristics

**Figure 1:**
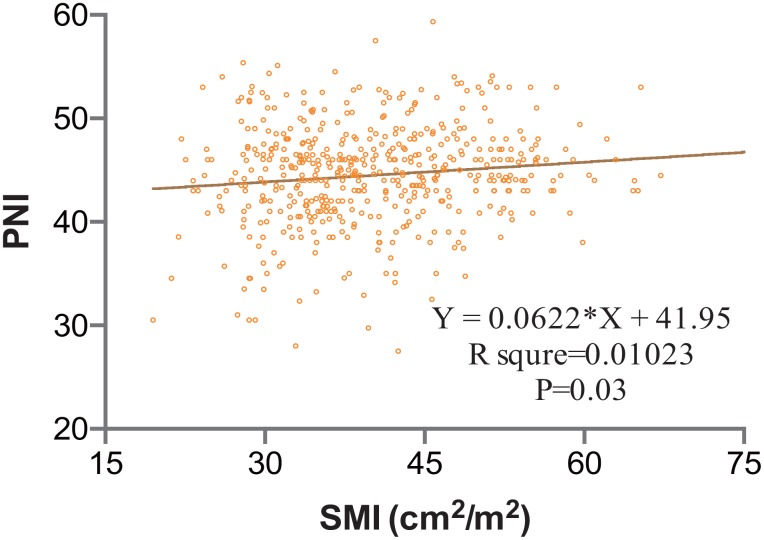
**Clinical Correlation between SMI and PNI; Value of R square for each relation was calculated.** R square was 0.01. Statistical significance was determined. SMI, skeletal muscle index; PNI, prognostic nutrition index.

Median follow-up duration was 24 months, ranging from 3 months to 77 months. Median BMI was 24.1 kg/m^2^ (ranging from 14.9 to 36.8 kg/m^2^), including males of 26.0 kg/m^2^ and females of 21.7 kg/m^2^ (P-value<0.001), while median SMI value of males has no statistical difference compared to females (40.5 cm^2^/m^2^ versus 39.0 cm^2^/m^2^, P-value=0.064). Linear regression relationship between SMI and PNI was determined in Figure 1 (P<0.05) and the R square for relation was 0.01.

**Figure 2:**
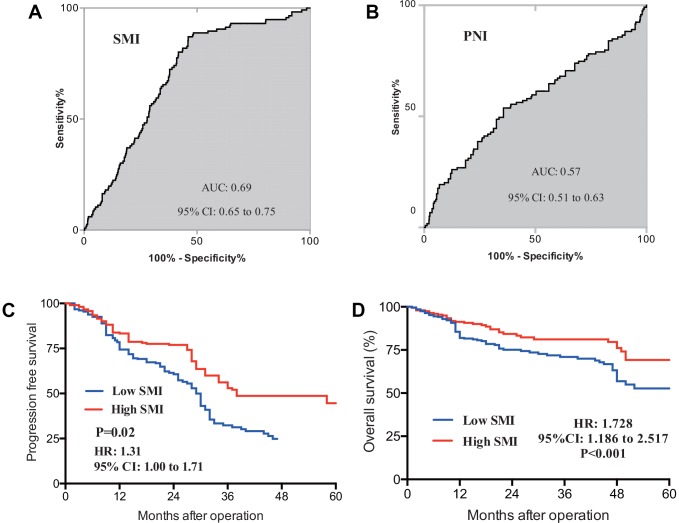
ROC curves for baseline SMI (**A**) and PNI (**B**) were constructed, AUC for SMI and PNI were separately 0.69 and 0.57; prognostic value of SMI in PFS (**C**) and OS (**D**) was also determined in CRLM patients, Patients with initial low SMI were found to have significantly shorter duration of PFS (P=0.002) and OS (P-value<0.001). ROC, receive operating curve; AUC, area under curve; SMI, skeletal muscle index; PNI, prognostic nutrition index; PFS, progression free survival; OS, overall survival.

When cut-off values for SMI were applied (43 cm^2^/m^2^ for males and 41 cm^2^/m^2^ for females), the cohort was divided into 309 (57.3%) and 230 (42.7%) patients with low and high SMI. Baseline low SMI was observed in 116 of 184 females (63.0%) and 193 of 355 males (54.4%) (P=0.05). Comparisons of clinical features of CRLM patients with low or high-SMI were shown in Table 2. Patients with low SMI had significantly smaller pre-albumin (P-value=0.01), smaller PNI (P-value=0.04) and shorter hospital stays (P-value=0.03) compared with those with high SMI. There was no difference in the value of transferrin, CRP, lymphocyte count, CEA and CA19-9. To identify the prognostic role of baseline SMI, ROC curves and Kaplan-meier analysis were performed. As is shown in Figure 2, time-dependent ROC curves were separately constructed to compare clinical value of baseline SMI and PNI. Area under curve (AUC) of baseline SMI and PNI were 0.69, 0.57 in Figure 2A and 2B, respectively. CRLM patients with low SMI was demonstrated to have shorter duration of PFS (Figure 2C, P-value=0.02, HR: 1.31; 95%CI: 1.00-1.71) and OS (Figure 2D, P-value<0.001, HR:1.728; 95%CI: 1.186-2.517) than their high SMI counterparts.

**Table 2:** Clinicopathological factors in CRLM patients with baseline different SMI levels.

	**Low SMI (n=309)**	**High SMI (n=230)**	**P-value**
Ages (years)	61.6±11.6	59.3±11.9	**0.01**
Gender (Females/Males)	116/193	68/162	0.05
SMI (cm^2^/m^2^)	33.6±4.4	48±6.0	**<0.001**
BMI (kg/m^2^)	22.8±4.2	25.6±4.4	**<0.001**
Albumin (g/dl)	38.5±5.0	38.8±6.7	0.48
Pre-albumin (g/dl)	0.12±0.05	0.15±0.04	**0.01**
Total bilirubin (mg/dl)	10.8±6.7	11.3±4.4	0.64
Platelet count (*10^3^/mm^3^)	213.3±88.0	219.1±87.6	0.45
Lymphocyte count	1.16±0.42	1.13±0.39	0.51
platelet/lymphocyte ratio	204.4±113.6	214.3±120.4	0.33
C-reaction protein	9.8±6.2	11.5±12.6	0.32
CRP/Alb ratio	0.27±0.44	0.35±0.71	0.14
CEA (ng/L)	52.0±118.8	54.3±132.4	0.86
CA19-9 (ng/L)	210.1±710.6	169.4±848.3	0.60
Prognostic nutritional index	40.2±5.4	44.1±7.8	**0.04**
Hospital stay (days)	13.7±8.2	11.7±6.9	**0.03**

Note: comparison of clinical characteristics between different SMI levels were determined by paired t-test.

### SMI as an indicator on treatment response and tumor progression

**Figure 3:**
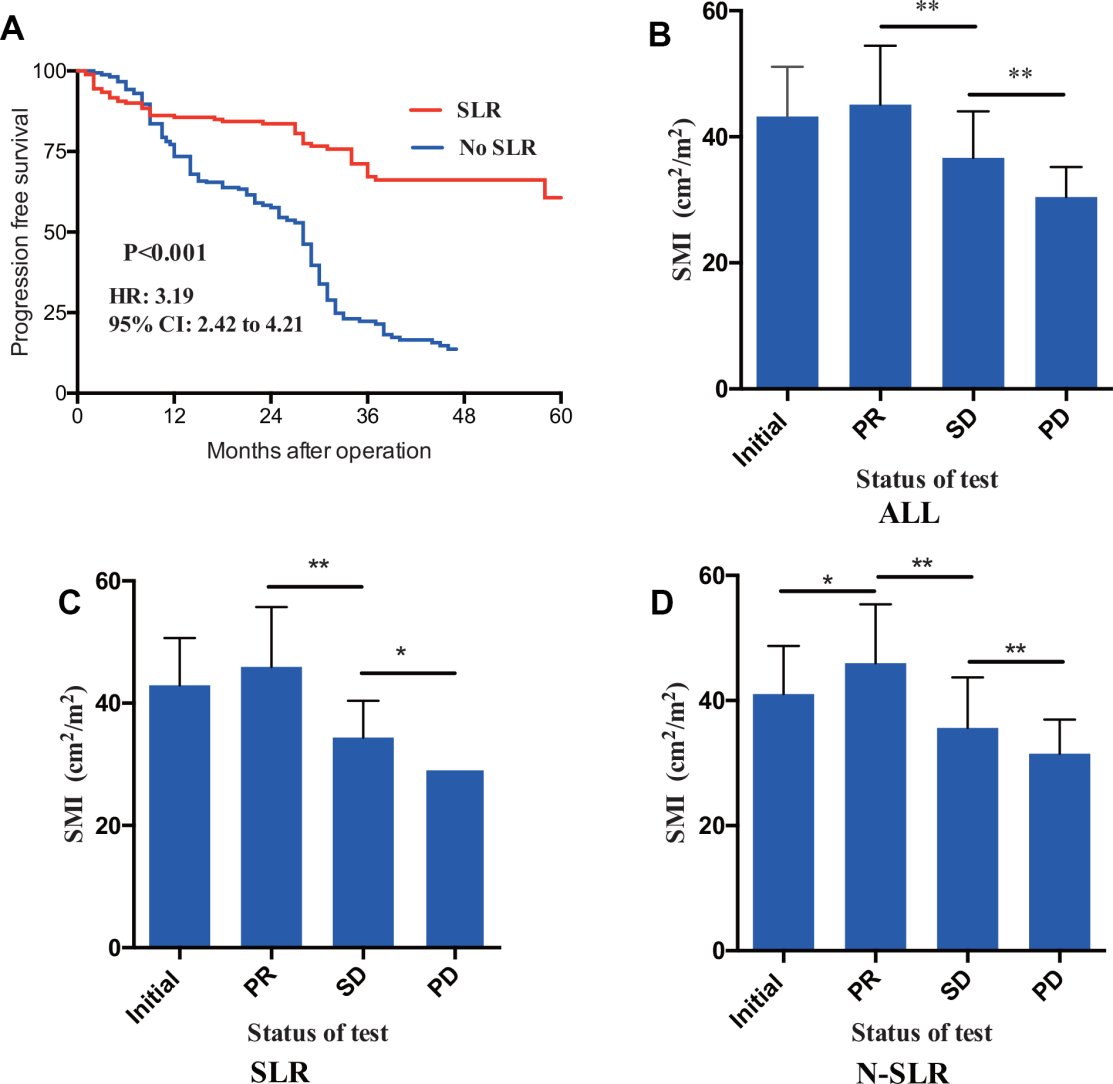
(**A**) Through Kaplan-meier analysis, patients received simultaneous liver resection (SLR) had statistically longer duration of PFS than patients received no SLR. Value of SMI were recorded in addition to therapeutic response. CRLM patients in the status of PR showed a larger SMI compared to SD and PD (**B**). Furthermore, fish exact tests were performed in SLR (**C**) cohort patients and N-SLR (**D**) cohort patients, respectively. PFS, progression free survival; CRLM, colorectal cancer liver metastasis; PR, partial response; SD, stable disease; PD, progressive disease.

CRLM patients was divided into two different cohorts based on whether simultaneous liver metastasectomy was performed: simultaneous liver resection (SLR) cohort and no simultaneous liver resection (N-SLR) cohort. Totally, SLR cohort patients had longer duration of PFS compared with N-SLR patients (Figure 3A, P-value<0.01, HR: 3.19; 95%CI: 2.42-4.21). Specifically, SLR cohort included 181 patients, occurrence or metastasis was observed in 50 patients (27.6%), while N-SLR cohort included 358 patients and occurrence or metastasis was observed in 194 patients (54.2%).

To compare indicative efficacy of SMI on therapeutic response, patients at the status of PR (namely during the treatment process had increased SMI, whereas patients regarded as having PD/SD had decreased SMI (loss of muscle volume) (Figure 3B, Fisher’s exact test, P-value<0.01). Table 3 included SMI value in initial diagnosis, PR, SD and PD status. Furthermore, a subgroup analysis was also performed to compared indicative role of SMI. In SLR cohort, median value of SMI at status of initial diagnosis, PR, SD and PD was separately 41.87cm^2^/m^2^, 46.89cm^2^/m^2^, 34.36 cm^2^/m^2^ and 28.55 cm^2^/m^2^ (P-value<0.01), whereas value of SMI at status of initial diagnosis, PR, SD and PD was 39.17 cm^2^/m^2^, 45.51 cm^2^/m^2^, 34.75 cm^2^/m^2^ and 30.47 cm^2^/m^2^ (P-value<0.001), which is also shown in Figure 3C and 3D.

**Table 3:** SMI values in different therapeutic response of CRLM patients.

**SMI (ALL)**	**Mean**	**Median**	**95% CI**	**P-value**
Status of test				P<0.001
Initial	43.20	42.94	42.47-43.92	
PR	45.09	45.22	44.28-45.91	
SD	36.66	36.13	36.02-37.30	
PD	30.43	29.93	29.81-31.05	
SMI (SLR)				
Status of test				P<0.001
Initial	42.93	41.87	42.15-43.71	
PR	45.92	46.89	44.98-46.87	
SD	34.40	34.36	33.82-34.98	
PD	29.31	28.55	28.57-30.04	
SMI (N-SLR)				
Status of test				P<0.001
Initial	41.05	39.17	39.95-42.16	
PR	45.97	45.51	44.80-47.15	
SD	35.64	34.75	34.63-36.64	
PD	31.51	30.47	30.83-32.19	

Note: comparison of SMI value between different treatment status were determined by paired t-test.

### 6 months’ loss of SMI is indicative for worse prognosis in CRLM patients

**Figure 4:**
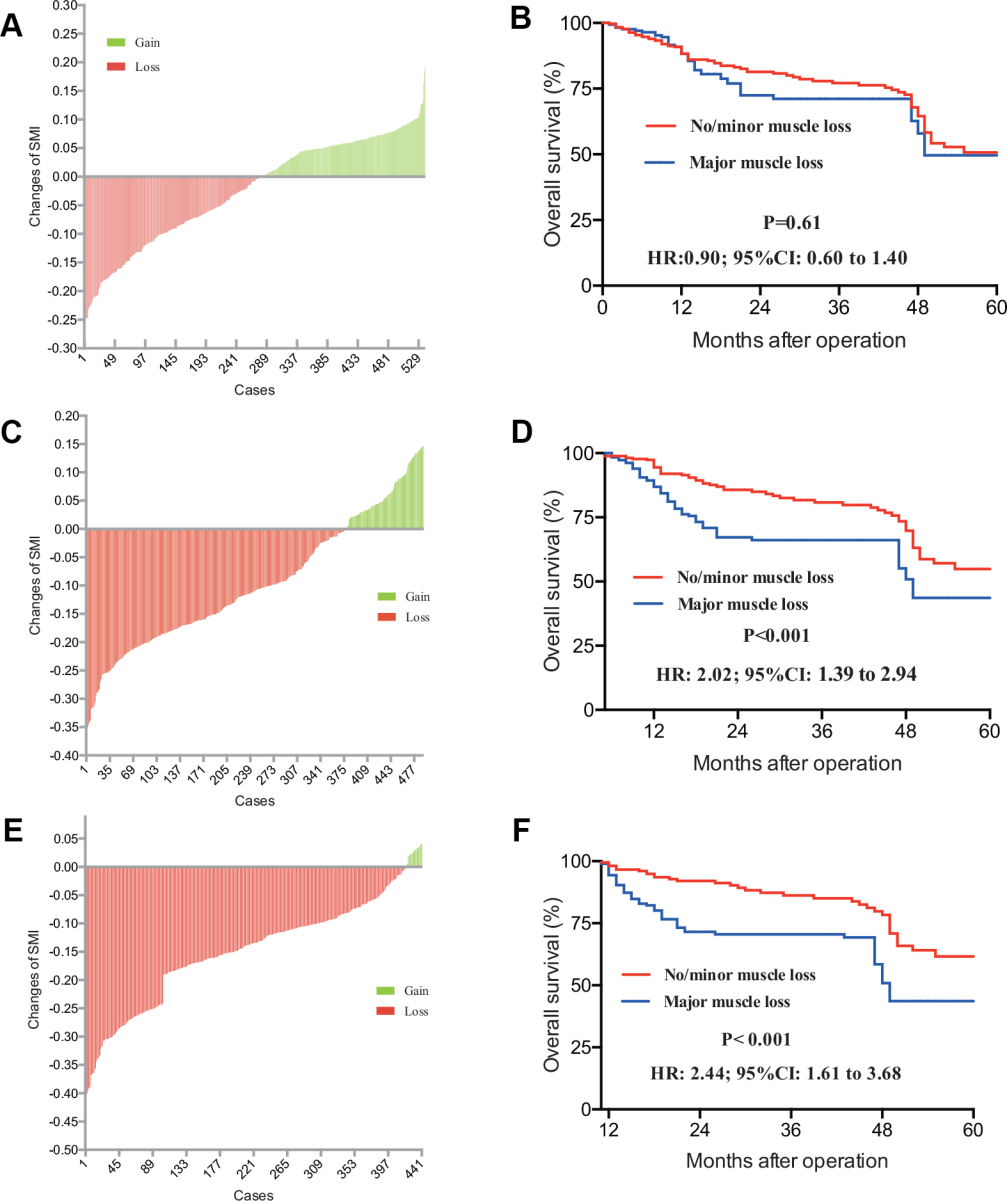
CRLM Patients with gain or loss of SMI was distributed in 3 months (**A**), 6 months (**C**) and 12 months’ (**E**) post-operation. Accordingly, prognostic value of loss of SMI with different post operative CT scan was determined. Patients with different SMI status from 3 months’ (**B**) scan haven’t demonstrated any survival difference (P=0.061), while patients with different SMI status 6 months’ (**D**) and 12 months’ (**F**) SMI results had different survival outcome (P-value<0.001). CRLM, colorectal cancer liver metastasis; SMI, skeletal muscle index; CT, computed tomography.

To explore the indicative clinical value of SMI changes in CRLM patients, further analysis was performed. Table 4 confers all changes of SMI and SMD in each period after the operation. Compared with the significant changes in muscle area (MA), our analysis showed that muscle density was not statistically changed after operation. For the change of MA, at the first postoperative evaluation of SMI based on CT scan (3 months after operation), median change of MA was -3.7% (Figure 4A) (skeletal muscle mass decreased by 3.7 percent), ranging from -25.6% to 19.4% (skeletal muscle mass increased by 19.4 percent). On the following 6 and 12 months postoperative CT scan, median change of MA was separately -8.8% (95% CI: -11.7% to -2.3%) and -10.1% (95% CI: -15.2% to -6.5%) (Figure 4C and 4E). It is reported that 8% decreased SMI was regarded as the cut-off value of major SMI loss [33]. Figure 4B demonstrated that CRLM patients with loss of SMI at the 3 months CT scan had not statistically different survival outcome with another SMI-stable group (P=0.61). As are shown in Figure 4D and 4F, 8% cut-off loss of SMI recorded from more than 6 months postoperative CT scan was regarded as a significant risk factor indicating worse prognosis in CRLM patients (P-value<0.001). Subgroup analysis was also performed for SLR cohort patients and N-SLR cohort patients, respectively (Supplementary Figure 2A–2F).

**Table 4:** Changes of muscle mass and density after operation (n=539).

**Variables**	**First scan**		**Second scan**		**Change 3 months post operation**			**Changes 6 months post operation**			**Changes 12 months post operation**		
	**Mean**	**SD**	**Mean**	**SD**	**Mean**	**95%CI**	**P-value**	**Mean**	**95%CI**	**P-value**	**Mean**	**95%CI**	**P-value**
SMA (cm^2^)	116.7	24.5	113.8	23.9	-3.7	-10.1 to 4.2	0.01	-8.8	-11.7 to -2.3	<0.001	-10.1	-15.2 to -6.5	<0.001
SMI (cm^2^/m^2^)	41.9	9.1	38.9	8.9	-3.7	-10.1 to 4.2	0.01	-8.8	-11.7 to -2.3	<0.001	-10.1	-15.2 to -6.5	<0.001
Male	43.2	8.2	39.2	8.6	-4.7	-15.1 to 7.0	<0.001	-9.9	-15.2 to -3.2	<0.001	-12.4	-18.8 to -6.2	<0.001
Female	39.5	10.1	37.1	9.3	-1.9	-3.8 to -0.1	0.01	-6.5	-8.9 to -1.7	<0.001	-8.8	-11.2 to -5.3	<0.001
SMD (HU)	34.4	8.1	33.2	5.4	-0.8	-1.2 to 0.3	0.45	-1.1	-1.8 to -0.2	0.18	-1.3	-2.0 to -0.6	0.13
Male	36.7	7.2	35.0	8.7	-1.2	-2.3 to -1.0	0.30	-1.3	-2.1 to -0.4	0.15	-1.5	-2.2 to -0.9	0.12
Female	30.3	8.8	29.1	3.2	-0.2	-1.1 to 1.2	0.83	-0.9	-1.4 to 0.1	0.42	-1.1	-1.8 to -0.4	0.51

**Abbreviations:** SMA, skeletal muscle area; SMD, skeletal muscle density; SMI, skeletal muscle index; HU, Hounsfield unit; SD, standard deviation.

### Prognostic efficacy of PNI in CRLM patients

**Figure 5:**
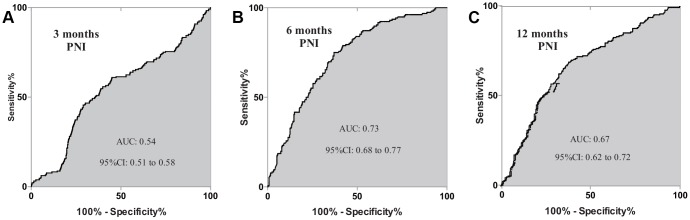
Prognosis dependent ROC curves of PNI were constructed in CRLM patients. AUC of 3 months, 6 months, 12 months were separately 0.54, 0.73 and 0.67. ROC, receive operating characteristics; PNI, prognostic nutrition index; CRLM, colorectal cancer liver metastasis; AUC, area under curve.

Since 3, 6 and 12 month PNI were also recorded, prognostic efficacy of PNI was also calculated. Figure 5 revealed that, for all patients, AUC of 3 months, 6 months and 12 months were separately 0.54, 0.73 and 0.67. Further analysis was also performed in both SLR and N-SLR group patients. As are shown in Supplementary Figure 2G–2L, 6 months PNI demonstrated more accurate prognostic value with a higher AUC in SLR (0.72) and N-SLR cohorts (0.73).

### Consistency analysis of SMI and PNI in resected CRLM patients

Totally, there is no statistical difference of median SMI between baseline and 3 months postoperative CT scan (Table 4), whereas 6 months and 12 months CT scan showed totally decreased SMI (P-value<0.001). As status of SMI and PNI in 6 and 12 months conferred prognostic significance, Kappa analysis of was made to compare the consistency among 3, 6 and 12 months. As is shown in Table 5, Kappa value of SMI and PNI between 6-month’s and 12-month’s cohort were 0.62 and 0.53 (P-value<0.001). In contrast, 3 months’ SMI and PNI were not statistically significantly consistent with 6 months’ and 12 months’ result.

**Table 5:** Kappa consistency analysis between different period CT scan and PNI examination.

**Months after operation**				**Kappa value**	**P-value**
3	6	No Loss of Muscle (n)	Loss of Muscle (n)		
No Loss of Muscle (n)		135	245	-0.22	P<0.001
Loss of Muscle (n)		100	59		
3	12	No Loss of Muscle (n)	Loss of Muscle (n)		
No Loss of Muscle (n)		48	298	-0.18	P<0.001
Loss of Muscle (n)		47	48		
6	12	No Loss of Muscle (n)	Loss of Muscle (n)		
No Loss of Muscle (n)		85	58	0.62	P<0.001
Loss of Muscle (n)		10	294		
Months after operation				Kappa value	P-value
3	6	Low PNI (n)	High PNI (n)		P<0.001
Low PNI (n)		112	234	-0.30	
High PNI (n)		102	43		
3	12	Low PNI (n)	High PNI (n)		P<0.001
Low PNI (n)		43	303	-0.21	
High PNI (n)		52	43		
6	12	Low PNI (n)	High PNI (n)		P<0.001
Low PNI (n)		78	66	0.53	
High PNI (n)		11	286		

### Combined test of post-operative SMI and PNI improve the prognosis prediction power in CRLM patients

**Figure 6:**
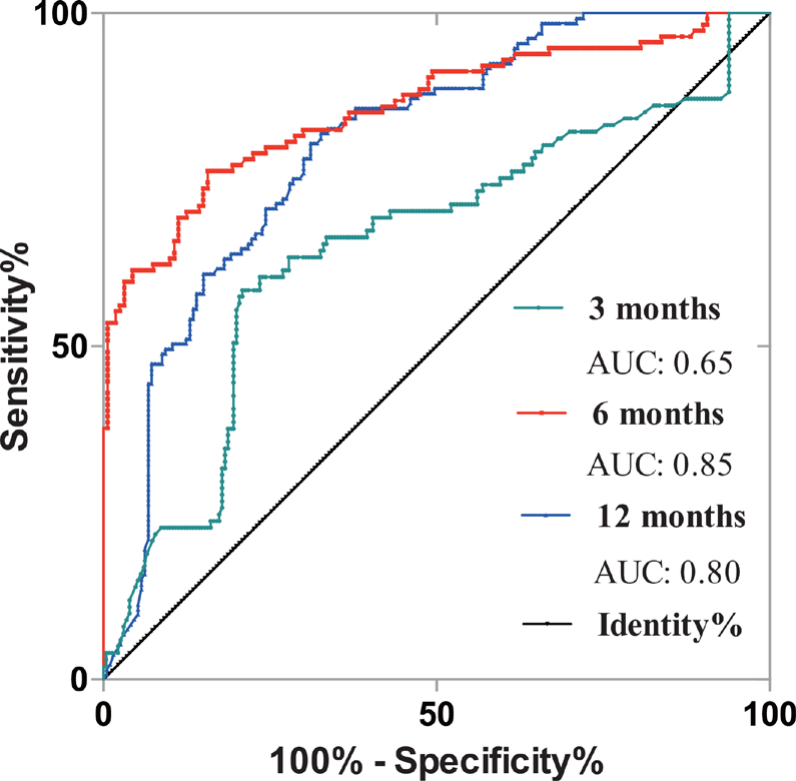
**Exploration of synergistic prognostic effect of PNI and SMI in CRLM patients.** 3-month’s, 6-month’s and 12-month’s results were analyzed. The AUC of 3-month, 6-month and 12-month were 0.65, 0.85 and 0.80. PNI, prognostic nutrition index; SMI, skeletal muscle index; CRLM, colorectal cancer liver metastasis; AUC, area under curve.

We further constructed the binary logistic regression model combining the tests of SMI and PNI. Figure 6 depicted prognostic potency of different postoperative period (3-, 6- and 12-month) with ROC curves. Furthermore, when the two biomarkers were combined using the binary logistic modeling, all three AUCs were significantly better than single SMI or PNI test. AUC of 3-month, 6-month and 12-month were separately 0.65, 0.85 and 0.80 (Figure 6).

## DISCUSSION

Currently surgery remains the cornerstone in the multimodal treatment of CRLM [4, 11, 28]. After operation was performed, effective assessment reflecting tumor progression and prognosis are warranted. Factors affecting cancer progression, treatment response and prognosis are multifaceted. Recently an increasing attention has been drawn to the correlation between cancer and nutritional status [12]; prognostication role of some nutritional markers such as PNI and BMI has been proven to be related with cancer [33, 13].

CT and MRI were the main methods evaluating therapeutic response. Beyond tumor assessment, CT still included other imaging information such as muscle volume and is not fully explored. In 2012, Lisa Martin [33] has reported that third lumbar vertebrae CT scan was the specific level reflecting skeletal muscle volume. These years, many reports concerning clinical value of SMI were identified in many cancers including CRC [11, 14–16]. However, clinical value of changes in SMI on resected CRLM patients was limited. Thus in this study we aim to explore the value of SMI recorded from L3 CT scan in CRLM patients after operation. Beyond SMI, combined effects of SMI and PNI were also explored in these patients.

We collected 539 CRLM patients from 2013 to 2016 who received resection of primaries companied with or without simultaneous liver metastasectomy. First, our data showed that patients with older age or larger BMI would had a larger SMI (P=0.01), which was consistent with notion that senescence was a key factor influencing muscle volume and nutritious status [9]. As skeletal muscle volume may be heterogeneous due to gender difference, exploration of relationship between SMI and sex was analyzed and there is no statistical difference on baseline SMI between males and females (P=0.05). This may be owing to the fact that overweight is one of high risk factors on CRC tumorgenesis [17–19]. Besides, indicative value of baseline body composition was explored. Univariate analysis and multivariable analysis conferred that patients with high SMI had significantly better survival outcomes compared with low-SMI cohort, which was in consistent with previous reports [20, 21]. In CRLM patients, previous studies evaluating potential prognostic factors in surgically treated patients with CRLM have focused on PNI, PLR, or CA19-9 individually [22, 23]. Present study also compared the difference of these factors in different baseline SMI cohorts. From our analysis, patients with lower baseline SMI had statistically smaller serum pre-albumin value (P=0.01), smaller PNI (P=0.04) and longer hospitalization days (P=0.03). These results demonstrated that some physiobiological markers were correlated with SMI [24, 25]. Two prognosis-dependent ROC curves were further separately constructed to compare predictive role of SMI and PNI. The results showed SMI (AUC: 0.69) had a good prognostic predictive power more than PNI (AUC: 0.57).

Tumor recurrence and metastasis were recognized as the most essential factors for survival. Here, we provide additional evidence that SMI was predictive for tumor progression. Comparing PFS from baseline high SMI cohort to Low SMI cohort, the median duration of PFS significantly decreased from 38 months to 29 months (P=0.002). Furthermore, patients in status of PD also had lower SMI compared with other status of the CT test, which demonstrating that SMI can consistently reflect the treatment response to some extent. Moreover, it must be noted that N-SLR subgroup analysis showed a higher SMI in PR status (P-value<0.001).

Changes of SMI were also recorded. In the present study, 3 different change trend charts were drawn to express the postoperative 3 months, 6 months and 12 months CT scan. Totally, skeletal muscle volume in CRLM patients is losing (Figure 4). Patients suffered from major SMI loss after 6 months from operation was regarded as the risk factor for overall survival, which demonstrated that 3-month’s status is not enough or accurate to judge the real postoperative nutritional conditions. To further confirm the result, kappa consistency analysis was performed among the 3, 6 and 12 months. Results have showed 6 months’ status had a relatively higher consistency with 12 months (kappa value of 0.62), whereas 3 months’ status was not consistent with either 6 or 12 months’ results. This may indicate that SMI tested in shorter postoperative duration may not reflect exact disease status.

PNI, a classic immune-nutritional marker [26], was reported to be prognosis relating [27]. In this paper, we found that 6 months recorded PNI showed a better prognosis reflection power than 3 months and 12 months’ results with an AUC of 0.73. Furthermore, we compared combined prognostication value of SMI and PNI in CRLM patients. Combined test of both markers can significantly improve prognosis predictive power than counting on merely one marker, besides, 6 months’ SMI and PNI demonstrated a higher AUC than 3 and 12 months.

However, there are some limitations of this study, including the retrospective design of the study, which may include selection bias, and the small sample size. We believe that well-designed statistical criteria can alleviate this problem. A large scale prospective, randomized controlled study may be warranted to strongly determine the prognostic value of SMI. Another limitation is that our study only focused on the CRLM. Some CRC patients with extra-hepatic metastasis were excluded in this study because bias caused by the anatomical variety can influence the statistical results. Furthermore, more detailed research works of the tumor indicators for prediction of CRC are still required in the future.

In summary, we found that SMI and PNI are good markers in patients with resected CRLM. Here we also proved the value of real-time monitoring of SMI in indicating therapeutic response during treatment, and 6-month’s changes of SMI and record of PNI revealed a more certain and accurate significance correlated with clinical prognosis. Furthermore, combined test of PNI and SMI could improve the prognosis prediction power in CRLM patients.

## MATERIALS AND METHODS

### Patients and characteristics

The study was approved by the Ethical Committee of Zhongshan Hospital, Fudan University. All included patients met the following criteria: 1) primary tumor resection with or without simultaneous liver metastasectomy of CRLM at Fudan University Zhongshan Hospital between January 2013 and December 2016; 2) available blood test records; 3) available follow-up information. The exclusion criteria were as follows: patients with infections, hematological disease, hyperpyrexia, and intestinal obstruction at test were excluded; patients with no available CT images and incomplete clinical and pathologic data were excluded. The type of surgical resection and the extent of lymph node dissection were selected according to Chinese colorectal cancer treatment guidelines [28, 29]. Tumor response including partial response (PR), stable disease (SD) and progressive disease (PD) were based on response evaluation criteria in solid tumors (RECIST 1.1) [30]. All patients provided written informed consent. Details of flow gram was demonstrated in Supplementary Figure 1.

### Preoperative anthropometric and blood-chemistry measurements

Physical status and preoperative laboratory values were obtained within 1 week prior to initiation of surgery. Body mass index (BMI) was calculated by dividing the body weight in kilograms by the square of the height in meters. CT was performed on an average of 4.2 days (range, 1–12 days) before surgical operation using a 320-slice multi-detector CT scanner (Aquilion ONE; Toshiba Medical Systems Corporation, Otawara, Japan) [31]. Skeletal muscle areas, including the psoas, erector spinae, quadratus lumborum, transversus abdominis, external and internal obliques, and rectus abdominis, were identified and quantified using -29 to 150 Hounsfield units. Specifically, CT images of less than 12-month’s post-operation were recorded as three cohorts (3-month cohort, 6-month cohort and 12-month cohort). SMI was calculated by normalizing skeletal muscle areas for height (cm^2^/m^2^) [32]. As is reported in 2013 [33], patients with SMI<41 cm^2^/m^2^ (women) and <43cm^2^/m^2^ (men) were significantly associated with low survival, thus we choose this value as a baseline cut-off value. PNI was calculated based on the serum albumin and total lymphocyte count, using the following equation: PNI=serum albumin (g/dL) + 5*total lymphocyte count (/mL) [34].

### Follow-up and statistics

The follow-up period was as follows: first follow-up was performed within 2–3 months after the baseline and subsequent follow-up cycles usually ranged from 3 to 6 months or even shorter which depended on the clinical situations and (or) tumor relapse or metastasis being suspected. Data of overall survival (OS) and relapse/metastasis time were also documented. Duration of OS was calculated from the date of baseline to the last follow-up or tumor-specific death. Progression free survival (PFS) was determined from the date of diagnosis to the date of progression under the regular follow-up.

Statistical analyses were performed using the SPSS statistical package (22.0; SPSS) and prism 6 (GraphPad Software, Inc., 2015). Mean changes in SMI were analyzed with paired t-tests. The correlations between continuous valuables were analyzed using Spearman rank correlation test. Predictive accuracy of tests was assessed by calculating AUC. Besides, Time-dependent cut-off values were determined when positive likelihood ratio (PLR) were the largest one [35, 36]. PLR was calculated as follows: PLR=sensitivity/(1–specificity). Binary logistic regression model of SMI and PNI were estimated based on SPSS 22.0. PFS and OS analyses were carried out using the Kaplan–Meier method and results were compared using a log-rank test. A multivariable Cox proportional hazards model predicting OS was performed using backward stepwise selection. Risk factors were expressed as the hazard ratio [HR, 95% confidence interval (CI)]. Statistical significance was defined as P-value less than 0.05.

## Supplementary Material

Supplementary Figures
