# Supplementary material for: Combined test of third lumbar skeletal muscle index and prognostic nutrition index improve prognosis prediction power in resected colorectal cancer liver metastasis
Source: Aging (Albany NY). 2019 Nov 22;11(22):10301–15. doi: 10.18632/aging.102457 (PMC6914437; doi:10.18632/aging.102457)
Supplement: Supplementary Figures [file aging-11-102457-s001..pdf]

SUPPLEMENTARY FIGURES

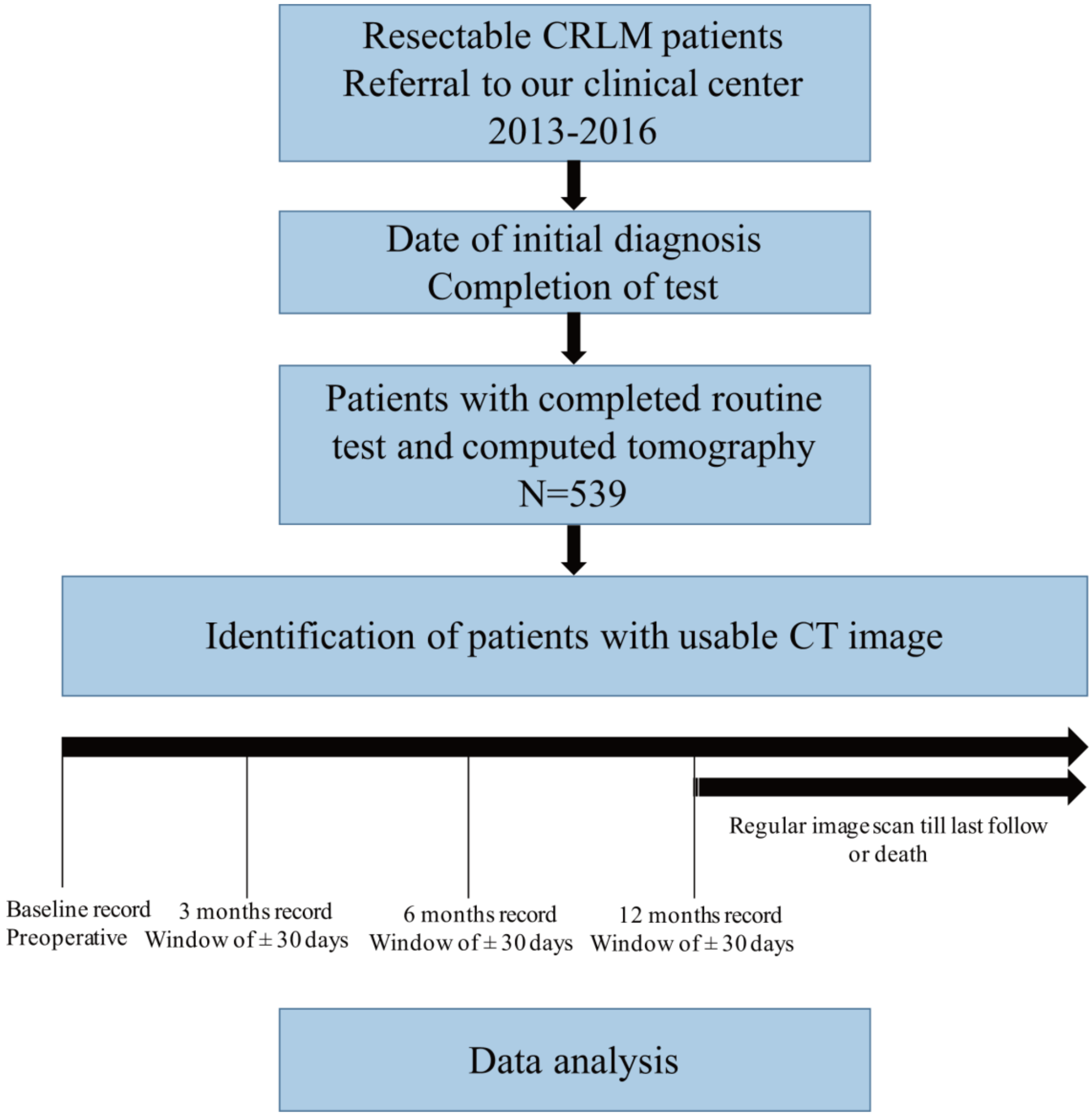

Supplementary Figure 1. Flow diagram of resected colorectal cancer liver metastasis patients enrolled from our institution.

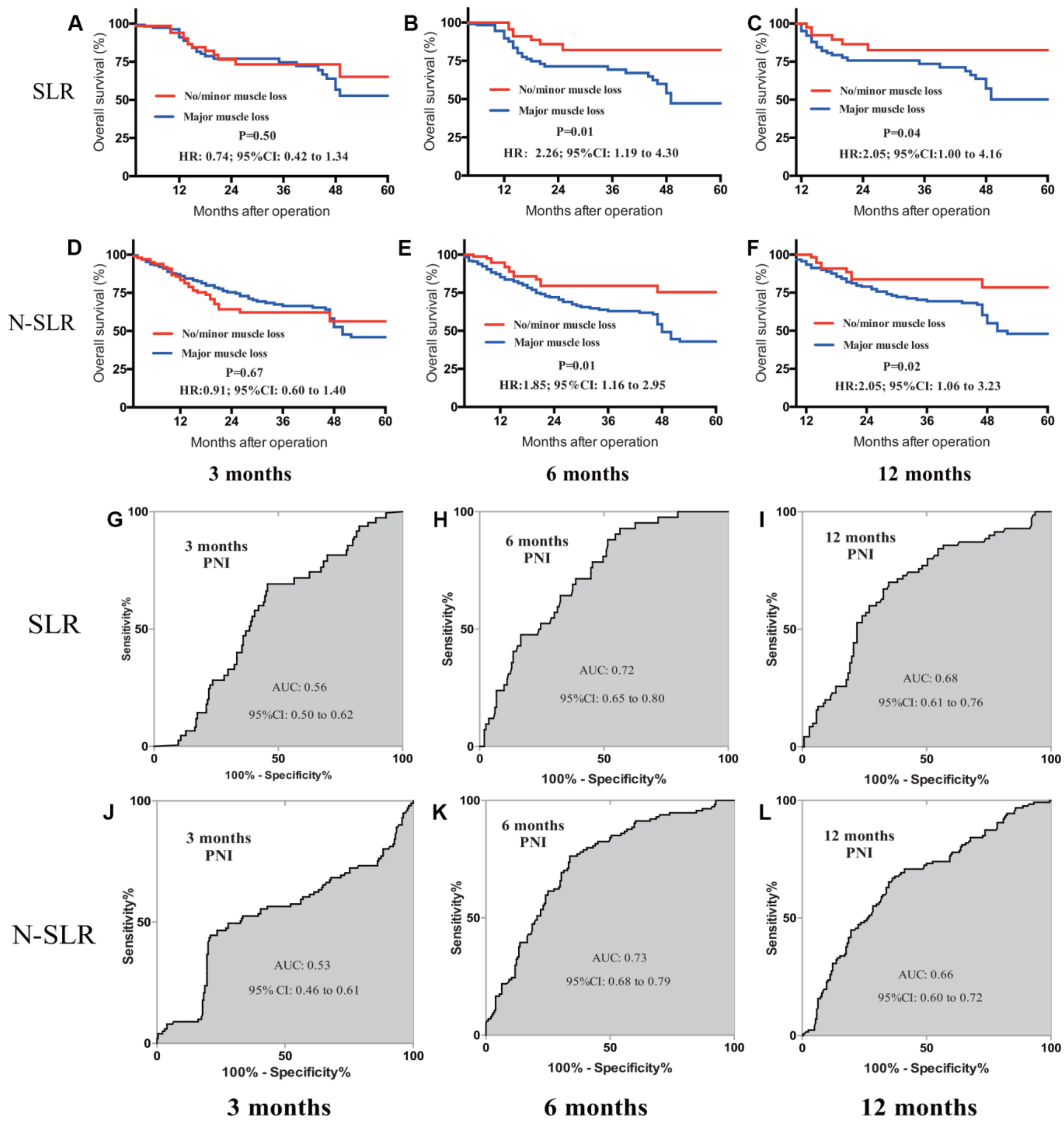

Supplementary Figure 2. **Subgroup prognostic analysis of SMI and PNI in CRLM patients.**
